# Endothelial cells are a source of Nestin expression in Pulmonary Arterial Hypertension

**DOI:** 10.1371/journal.pone.0213890

**Published:** 2019-03-18

**Authors:** Aneel R. Bhagwani, Schuyler Hultman, Daniela Farkas, Rebecca Moncayo, Kaivalya Dandamudi, Arsema K. Zadu, Carlyne D. Cool, Laszlo Farkas

**Affiliations:** 1 Department of Internal Medicine, Division of Pulmonary Disease and Critical Care Medicine, Virginia Commonwealth University, Richmond, VA, United States of America; 2 Department of Pathology, University of Colorado at Denver, Denver, CO, United States of America; Stanford University, UNITED STATES

## Abstract

Uncontrolled proliferation of endothelial cells is essential to the pathogenesis of pulmonary arterial hypertension (PAH). Both proliferation and cytoskeleton reorganization are associated with upregulation of the intermediate filament protein Nestin. Recently, accumulation of Nestin-expressing cells was found in pulmonary vascular lesions of PAH patients. The goal of this study is to determine if Nestin expression contributes to endothelial proliferation in pulmonary arterial hypertension, using both lung tissues and endothelial cells. Here we found that endothelial cells from complex and plexiform lesions of PAH patients expressed Nestin. These Nestin^+^ cells further stained positive for the angiogenic factors CXC chemokine ligand 12 and Wnt1. Likewise, in the chronic hypoxia/SU5416 animal model of pulmonary hypertension, Nestin^+^ endothelial cells were found in occlusive pulmonary vascular lesions. *In vitro*, both growing rat and human lung endothelial cells expressed Nestin protein. When Nestin was overexpressed in endothelial cells (both rat and human), Nestin overexpression promoted proliferation and expression of CXC chemokine ligand 12. Nestin overexpression further increased angiogenic tube formation *in vitro*. Conclusions: We found increased Nestin expression from endothelial cells of occlusive lung vascular lesions in severe pulmonary hypertension. Elevated Nestin expression likely contributes to unchecked pulmonary vascular proliferation and angiogenesis, possibly *via* induction of CXC chemokine ligand 12. Additional studies are required to determine whether targeting Nestin would be beneficial to treat PAH.

## Introduction

Pulmonary arterial hypertension (PAH) is a devastating and deadly condition characterized by a progressive occlusive arteriopathy in the lung. The extent of this remodeling in pulmonary arteries ranges from neointima formation and increased muscularization to complex plexiform lesions [1,2]. Today, patients with PAH have an improved prognosis due to pulmonary vasodilators. However, these treatments do not sufficiently target the occlusive arteriopathy in pulmonary arteries [3,4]. To improve upon existing therapeutic strategies, we need to better understand the intricacies of this pulmonary arteriopathy [2]. To date, we know that endothelial cells (ECs) and pulmonary artery smooth muscle cells (PASMCs) from PAH patients are hyper-proliferative [5,6]. Further, some studies have shown changes in pathways that regulate endothelial cell growth [6,7]. One current concept suggests that initial endothelial apoptosis leads to selection of these hyperproliferative ECs by clonal selection of surviving, apoptosis-resistant ECs [8]. Aberrant proliferation, apoptosis-resistance and clonal expansion are also typical features of cancer stem cells [9]. Hence, Lee *et al*. have argued that these apoptosis-resistant ECs may be derived from endothelial-like stem cells [8]. This helps to explain the expression of stem cell markers in the vascular lesions from PAH patients and rat models of pulmonary hypertension (PH) [10,11].

Recently, it was shown that pulmonary arteries from PAH patients express high levels of the type VI intermediate filament protein Nestin [12]. Neuronal stem cells, mesenchymal stem cells, and angiogenic endothelium all express this filament [13–17]. Nestin has an important function to promote self-renewal and proliferation in stem cells [13,18–20]. It is interesting that endothelial expression of Nestin is restricted to ECs undergoing proliferation and angiogenesis [16,21,22].

We hypothesized that abnormal endothelium in PAH pulmonary arteries expresses more Nestin and that overexpression of Nestin promotes proliferation and angiogenesis in lung ECs. We found Nestin^+^ ECs in the remodeled pulmonary arteries of PAH patients. These Nestin^+^ cells further stained positive for angiogenic factors, such as CXC chemokine ligand 12 (CXCL12) and Wingless-type MMTV integration site family, member 1 (Wnt1) [23,24]. We also found an accumulation of Nestin^+^ ECs in remodeled pulmonary arteries from chronic hypoxia and SU5416 (cHx/Su) rats with severe PH. To identify the role of Nestin in lung endothelium, we show that transient overexpression of Nestin increases CXCL12 expression, endothelial proliferation and angiogenesis *in vitro*. Hence our findings demonstrate a role for an increased level of Nestin expression in the endothelial proliferation and angiogenesis of PAH.

## Materials and methods

### Human tissue samples

Human de-identified lung tissue samples were obtained from the Department of Pathology, University of Colorado Denver. Formalin-fixed and paraffin-embedded lung tissue samples were sectioned at a thickness of 5 μm at the Department of Pathology, University of Colorado Denver. The collection of human tissue samples was approved by the local institutional research ethics board at the University of Colorado Denver in conformity with ethical guidelines of the Declaration of Helsinki of 1975, as revised in 1983. Informed consent was waived by the review board. The use of de-identified tissue samples was approved as non-human subjects research by the Office of Research Subjects Protection at Virginia Commonwealth University. The subject characteristics are provided in Table 1.

**Table 1:** Subject/Patient characteristics for the histological studies.

Group	Subject #	Gender	Age [years]	Diagnosis
**control**	1	Male	69	Donor lung
	2	Female	32	Donor lung
	3	Male	50	Resection margin, adenocarcinoma
**iIPAH**	4	Female	30	iPAH
	5	Female	40	iPAH
	6	Male	23	iPAH
	7	Female	29	iPAH
	8	Female	53	iPAH
	9	Male	40	iPAH

### Animal experiments

All animal experiments were approved by the VCU institutional animal care and utilization committee (protocol # AD10000821) and were performed according to the NIH Guide for the Care and Use of Laboratory Animals (8^th^ edition, 2011). cHx/Su-induced severe PH was established in male Sprague Dawley rats (Envigo, Indianapolis, IN) as described previously and animals were euthanized by exsanguination at the indicated time points under anesthesia with Ketamine (100 mg/kg) and Xylazine (15 mg/kg) (Henry Schein, Melville, NY) after invasive hemodynamic measurements to obtain right ventricular systolic pressure (RVSP) [25–27]. SU5416 was obtained from Sigma-Aldrich (St. Louis, MO). All efforts were made to minimize suffering. Lung and heart were removed *en bloc*, and the right lung was snap frozen for molecular biology studies. For EC isolation, lungs from naive rats were used to obtain a lung single cell suspension as previously described [26]. For histology, the left lung was inflated with 0.5% low-melting agarose (20 cmH_2_O) and formalin-fixed (48 h), then paraffin-embedded for immunofluorescence (IF) staining. Naïve control animals were kept at room air. Animals housed under conditions of cHx or SU5416 alone were housed as described previously and used as additional controls [26].

### Histology

Paraffin-embedded and formalin-fixed rat lung and heart tissue was sectioned at a thickness of 3 μm. Serial sections of human lung tissue were obtained from the Department of Pathology, University of Colorado Denver.

Immunohistochemistry (IHC), double and triple immunofluorescence (IF) stainings were performed as previously published by us [25,26]. The following primary antibodies were used: α-smooth muscle actin (SMA) (M0851, DAKO, Carpinteria, CA, dilution 1:200), CD31 (LS-NBP1-49805, Lifespan Biosciences, Seattle, WA, dilution 1:20), CXCL12 (ab89321, Abcam, Cambridge, MA, dilution 1:10), human Nestin (556309, BD Biosciences, dilution 1:50), human Nestin (LS-B51, Lifespan Biosciences, dilution 1:50), rat Nestin (ab93666, Abcam, dilution 1:10), rat Nestin (Cell signaling #4760, dilution 1:400), proliferating cell nuclear antigen (PCNA, #2586, Cell Signaling Technologies, Danvers, MA, dilution 1:100) vascular endothelial (VE)-cadherin (LS-C313199, Lifespan Biosciences, dilution 1:100), von Willebrand Factor (vWF) (A008202, DAKO, dilution 1:500), vWF (MA5-14029, Invitrogen, dilution 1:100) and Wnt1 (ab91191, Abcam, dilution 1:20). Secondary antibodies conjugated with Biotin, followed by horse radish peroxidase conjugated streptavidin and 3,3’-diaminobenzidine staining reaction, were used for IHC detection. Secondary antibodies conjugated with AF488, 594 and 647 were used for IF detection. For all IHC and IF stainings, controls with unspecific IgG were run in parallel with each staining batch.

### Confocal microscopy

Confocal microscopy was performed with a Zeiss LSM 700 upright laser scanning confocal microscope system housed in the VCU Department of Anatomy and Neurobiology Microscopy Core Facility.

### Quantification of IHC and IF stainings

For quantification of IHC, the number of positive cells and total cells were enumerated in 10 randomly acquired pulmonary arteries (magnification 400×) per animal by a blinded investigator as previously described using the cell counter plugin for Fiji/ImageJ [28]. The average fraction of positive cells vs. total cells per each animal was used for statistical analysis.

For quantification of double and triple IF stainings, 10 randomly selected pulmonary arteries were acquired from each lung tissue specimen at a magnification of 200× with a Zeiss LSM 700 laser scanning confocal microscope using specific filters for DAPI, AlexaFluor 488 and 594 or 647 (double IF), or DAPI, AlexaFluor 488, 594 and 647 (triple IF). The numbers of single and double positive cells per pulmonary artery (including lumen/lumen-occluding cells, cells in intima, media, adventitia and perivascular infiltrate), and the total number of cells were counted in the assembled multichannel image using the cell counter plugin of Fiji/ImageJ by an investigator blinded to the subject groups [28]. In addition, the blood vessels were categorized into the N/M group that includes both non-muscular and muscular pulmonary arteries, and the C/P group that summarizes pulmonary arteries with plexiform and concentric lesions. The statistical analysis was performed from the average fraction (double/triple positive cells vs. total cells) for each patient after categorization per vessel type/remodeling category and patient group, using the average fraction for each PAH patient in N/M and C/P categories as n = 1 (controls N/M only, n = 1).

### Quantitative real-time PCR

mRNA was extracted with the miRNeasy Mini Kit (Qiagen, Valencia, CA) according to manufacturer’s instructions. Reverse transcriptase reaction was performed according to established standard protocols. In brief, after DNAse I treatment, 1 μg of RNA was transcribed with random hexamer primers, deoxy nucleotides and MultiScribe RT (Life Technologies, Grand Island, NY). The following cycling program was used: 10 min at 25°C, followed by 120 min at 37°C, then by 5 min at 85°C.

For quantitative real-time PCR, the following QuantiTect Primer Assays (Qiagen) were used: *Rattus norvegicus*: *Nes* (QT00376922), *B2m* (QT00176295). For mouse Nestin, we used the following KiCqStart primer (Sigma Aldrich): *Mus musculus*: *Nes (*M_Nes1). Human primers: KiCqStart primer: H_PCNA_1, H_CXCL12_1, H_WNT1, H_TBP_1. The amplification was performed using Roche Lightcycler 480 (Roche Diagnostics, Indianapolis, IN), Stratagene Mx3000P (Agilent Technologies, Santa Clara, CA) and BioRad CFX 384 (BioRad, Hercules, CA), using the SYBR green master mix (Applied Biosystems). The cycling conditions were as follows: Preincubation for 15 min at 95°C, then Amplification (45 cycles with 15 sec at 94°C, 30 sec at 55°C and 30 sec at 72°C each). The values were calculated according to the mathematical model published by Pfaffl M [29] by normalization against *B2m* (rat) and *TBP* (human) as housekeeping gene. Values were expressed as n-fold of control samples. When a sample did not induce amplification (AdDL70 controls for overexpression of mouse Nestin in non-murine cells), the result was recorded as “0” for statistical analysis.

### Isolation of rat lung endothelial cells (ECs)

Rat lung ECs were isolated from lung single cell suspensions of naive male Sprague Dawley rats (Envigo). Rat lungs were removed, and a single cell suspension was prepared from the peripheral lung tissue using a modification of the protocol by van Beijnum *et al*. [30]. In brief, tissue was minced into <1mm^3^ pieces, and digested in a solution of 0.1% collagenase II and 2.5 U/ml dispase solution (both from Thermo Fisher Scientific, Waltham, MA) for 30 min at 37˚C. Then, tissue pieces were incubated with 0.1% DNase (Sigma-Aldrich, St. Louis, MO) for 30 min at 37˚C. CD31^+^ cells were obtained by immunomagnetic sorting using magnets and the “Any Species positive selection” kit from Stem Cell Technologies (Vancouver, BC) and CD31 antibody from R&D Systems (FAB3628). ECs were cultured on type I collagen coated dishes with EGM-2MV medium (Lonza, Walkersville, MD) and characterized by flow cytometry to identify EC markers CD144 (VE-cadherin, bs-0878R, Bioss antibodies, Woburn, MA) and vascular endothelial growth factor receptor 2 (VEGFR2, bs-10412R, Bioss). Rat lung ECs were used in passages 2–4. Expression of myeloid/hematopoietic markers CD133 (bs-0209R, Bioss) and CD11b/c (554862, BD Biosciences) was excluded by flow cytometry.

### Culture of human lung microvascular endothelial cells (HLMVECs)

HLMVECs were obtained from Lonza clonetics (CC-2527) and expanded in EGM-2MV (Lonza). HLMVECs were used in passages 3–7. For protein isolation, HLMVECs were seeded in 10 cm diameter cell culture dishes and grown to subconfluency.

### Culture of human pulmonary artery endothelial cells (PAECs)

Control human pulmonary artery ECs (PAECs) were obtained from the Pulmonary Hypertension Breakthrough Initiative (PHBI) and cultured in endothelial growth medium 2 (EGM-2, Promocell, Heidelberg, Germany). PAECs were used in passages 3–7. To arrest cell growth, PAECs were serum-starved by culturing in endothelial basal medium without serum and growth factors for 24h. A control group was grown in complete EGM-2.

### Adenovirus-mediated overexpression of Nestin and cell growth analysis

Rat lung ECs and human PAECs were transfected with 25–50 multiplicity of infections (MOI) of AdNes (adenovirus encoding murine Nestin, Vector Biolabs, Malvern, PA) or AdDL70 (empty control adenovirus without gene insert, courtesy of Dr. Martin Kolb, McMaster University) as previously published [31]. The virus was removed after 16h and the cells were removed after a total time of 72 hours for measuring transgene expression by qRT-PCR. For proliferation analysis, cells were pulsed with 10 μM 5-bromo-deoxyuridine (BrdU) for the final 4 hours. The cells were then fixed, permeabilized, DNAse treated, stained with APC-labeled anti-BrdU antibody (clone BU-1, R&D Systems) and labeled with 7-aminoactinomycin D (7-AAD, BD 559925). The cells were analyzed with a FACSCanto II flow cytometer (BD Biosciences) and FlowJo Software (FlowJo, LLC).

### Protein isolation and Western blot

Protein was then isolated using RIPA buffer lysis as previously described [26,32]. Western blots were prepared and stained with anti-cleaved caspase-3 antibody (#9661, Cell Signaling Technology), anti-CXCL12 antibody (#3530, Cell Signaling Technology), anti-Nestin antibody (556309, BD Biosciences and ABD69, Millipore Sigma, Burlington, MA), anti-PCNA (#2586, Cell Signaling Technologies), anti-α-tubulin antibody (#2125, Cell Signaling, loading control) and anti-β-actin antibody (A5441, Millipore Sigma, loading control) as previously described [26,32].

### Angiogenesis assay

Human PAECs grown in T75 tissue flasks were transfected with 50 MOI of AdNES or AdDL70, or not transfected (untreated control). The virus was removed after 16h, and after 48h, the cells were trypsinized and seeded in Ibidi μ angiogenesis slides (Ibidi, Planegg, Germany). For the seeding, Ibidi μ chambers were first filled with 10 μl of ice-cold matrigel (Corning, Corning, NY), which was solidified at 37°C, and then 50 μl of PAEC suspension (2×10^5^ cells per ml) were added. The cells were incubated for 20h and stitched images of each well were taken with 4x objective and an Olympus IX70 microscope and Olympus XM10 camera with cellSens Dimension software (all Olympus, Waltham, MA). After selecting the central rectangular area from each image, quantification was done using the angiogenesis analyzer plugin for Fiji.

### Statistical analysis

Data are presented as mean+SEM. Two Groups were compared with t-test or Mann-Whitney test (non-parametric data) or Kruskal-Wallis test followed by multiple comparison according to Dunn or Benjamin, Krieger and Yekutieli (>2 groups). Statistical analysis and graphs were done with GraphPad Prism 6.0 (GraphPad Software, LaJolla, CA). *P*<0.05 was considered significant.

## Results

### Nestin expression in the pulmonary vascular lesions of patients with severe PH

**Fig 1:**
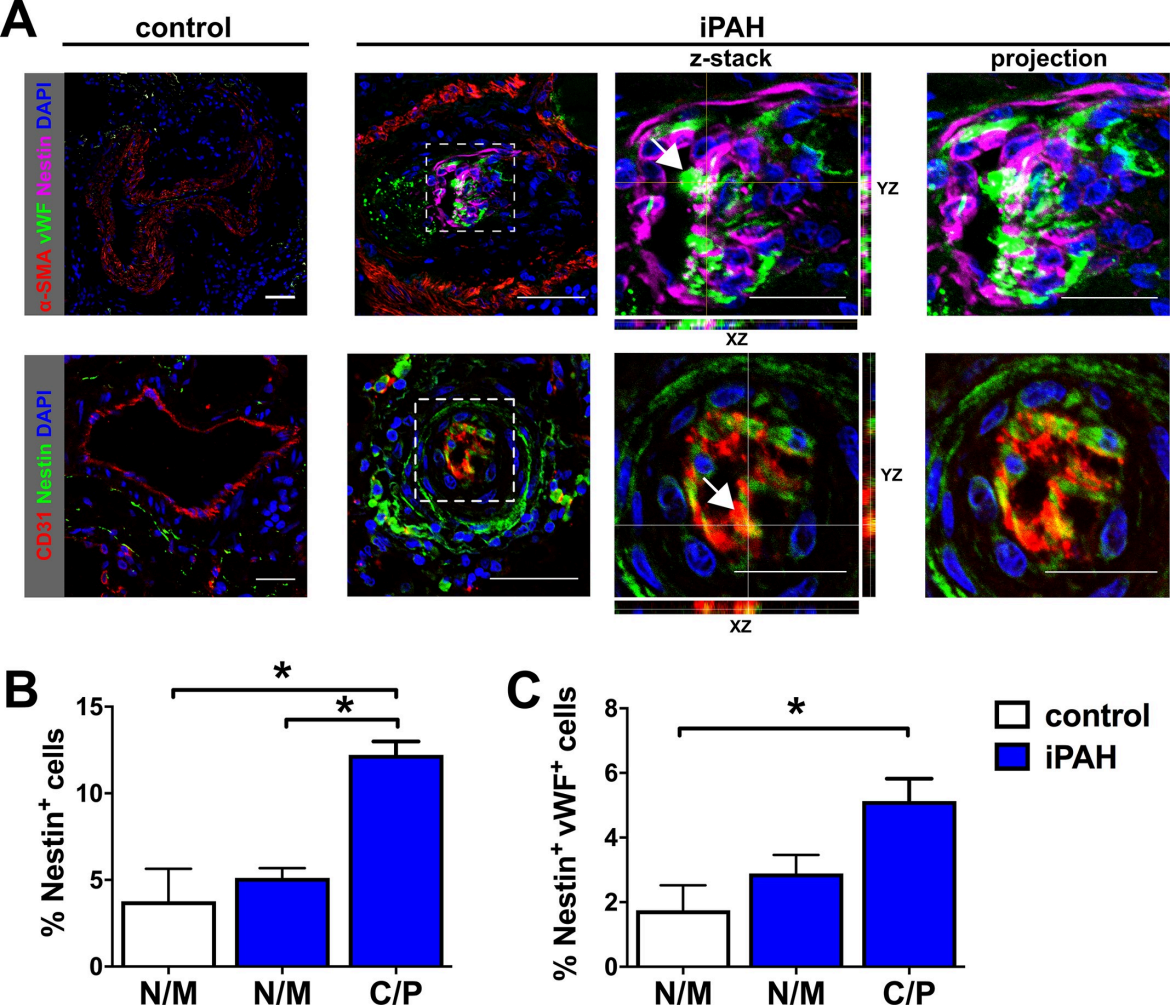
Endothelial cells are a source of Nestin expression in the remodeled pulmonary arteries from iPAH patients. (**A**) Representative merged immunofluorescence (IF) images of optical sections and Z-stacks obtained by confocal microscopy show Nestin/α-SMA/vWF and Nestin/CD31. Note that many Nestin^+^ cells were also vWF^+^ (overlap of green and magenta pseudocolors, shown in white—upper row) or CD31^+^ (overlap of red and green pseudocolors, shown in yellow—lower row) in the pulmonary arteries from iPAH patients (arrows). Nestin staining was infrequent in control lung tissue (no pulmonary vascular disease). For the iPAH group, the left image shows an overview of the pulmonary artery/vascular lesion. The center image demonstrates the area indicated by a dotted box in more detail including orthogonal views of the z-Stack in XZ and YZ directions. The image on the right shows a projection of all z-stack images. The thin yellow and white lines indicate the location of reslicing on the X-, Y- and Z-axis. Arrows show representative Nestin^+^ vWF^+^ and Nestin^+^ CD31^+^ cells. Scale bar: 50 μm (overview images), 25 μm (detail images). Nuclear counterstaining with DAPI. Fluorochromes and pseudocolors: Nestin [AF647 (magenta), AF488 (green)], α-SMA [AF594 (red)], vWF [AF488 (green)], CD31 [AF647 (red)]. (**B-C**) Quantification of the fraction of Nestin^+^ cells (B) and Nestin^+^ vWF^+^ (C) in the pulmonary arteries/lung vascular lesions. N/M: non-muscularized/muscularized pulmonary arteries, C/P: pulmonary arteries with concentric or plexiform lesion. Note that there are two N/M groups: white N/M bars represent controls and blue N/M bars represent iPAH N/M vessels. Graphs in (B-C) demonstrate analysis using average for each patient in each group. Each bar represents the mean+SEM of the average fraction of positive cells in pulmonary vessels categorized according to type of vessel remodeling (N/M or C/P) per patient. N numbers used for statistics were the numbers of patients: controls: N/M n = 3, iPAH: N/M and C/P n = 6. *P<0.05 (Kruskal-Wallis). The total number of pulmonary arteries: control: N/M n = 30; iPAH: N/M n = 41; C/P n = 19.

**Fig 2:**
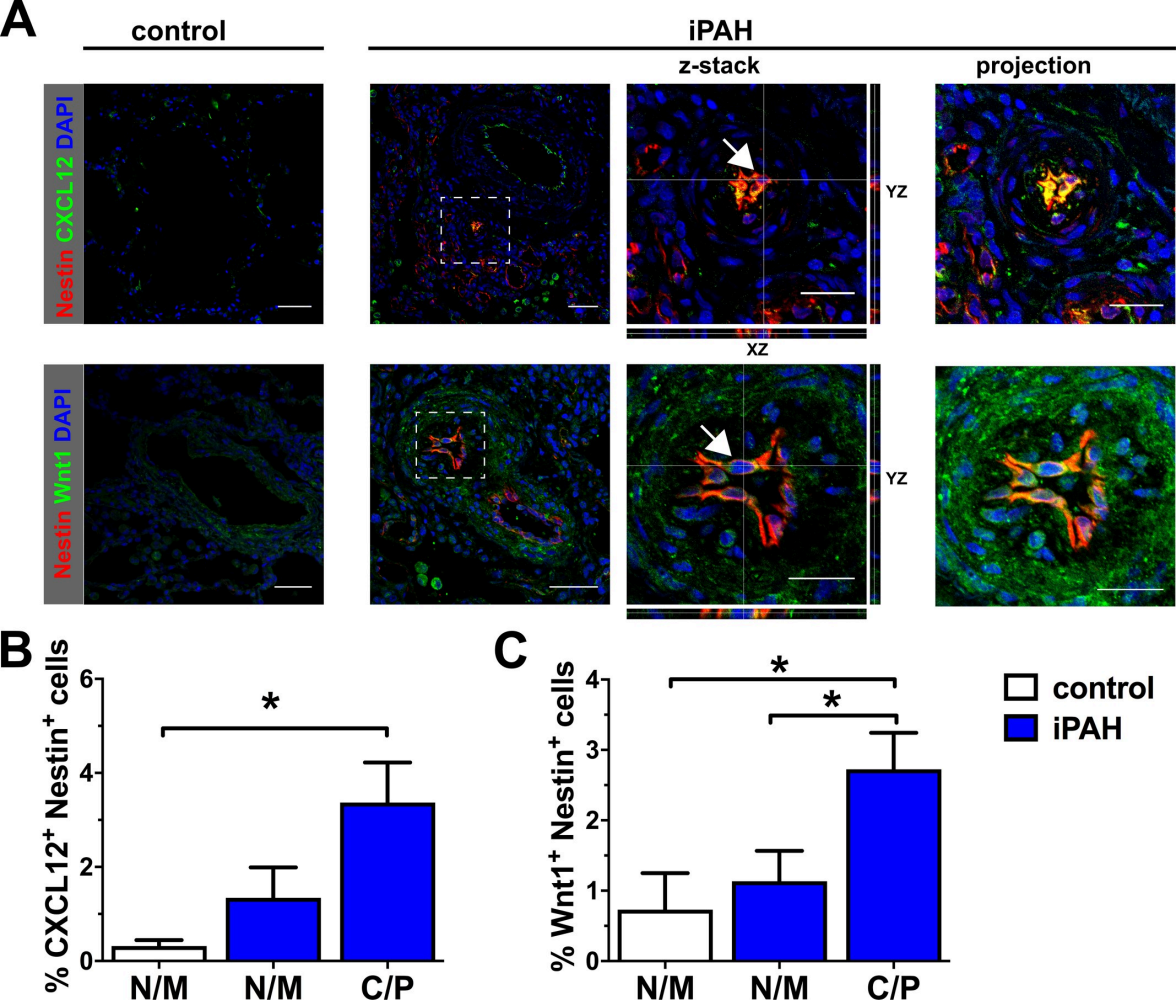
Nestin^+^ cells express angiogenic factors in iPAH pulmonary artery lesions. (A) Representative merged images of optical sections and z-stacks obtained by confocal microscopy show double IF stainings for Nestin/CXCL12 and Nestin/Wnt1. CXCL12 expression localized to pulmonary arterial lesions in iPAH lungs. It further co-localized (yellow) with Nestin in the vascular lesions (arrow). Control vessels had only scattered CXCL12 staining. Nestin^+^ Wnt1^+^ cells (yellow) were present in the pulmonary arterial lesions from iPAH patients (arrow). Nestin staining was absent in a control pulmonary artery. For the iPAH group, the left image is an overview of the pulmonary artery/vascular lesion. The center image demonstrates the area indicated by a dotted box in more detail. It also shows orthogonal views of the Z-Stack in XZ and YZ directions. The image on the right is a projection of all z-stack images. The thin white lines indicate the location of reslicing on the X-, Y- and Z-axis. Arrows show representative Nestin^+^ CXCL12^+^ and Nestin^+^ Wnt1^+^ cell. Scale bar: 50 μm (overview images), 25 μm (detail images). Nuclear counterstaining with DAPI. Fluorochromes and pseudocolors: Nestin [AF647 (red)], CXCL12 [AF488 (green)], Wnt1 [AF488 (green)]. (B-C) Quantification of the fraction of CXCL12^+^ Nestin^+^ cells (B) and Wnt1^+^ Nestin^+^ cells (C) in the pulmonary arteries/lung vascular lesions. N/M: non-muscularized/muscularized vessels, C/P: concentric or plexiform lesion. Note that there are two N/M groups: white N/M bars represent controls and blue N/M bars represent iPAH N/M vessels. Graphs in (B-C) demonstrate analysis using average for each patient in each group. Each bar represents the mean+SEM of the average fraction of positive cells in pulmonary vessels categorized according to type of vessel remodeling (N/M or C/P) and group (control vs. iPAH) per patient. N numbers used for statistics were the numbers of patients: controls: N/M n = 3, iPAH: N/M and C/P n = 6. *P<0.05 (Kruskal-Wallis). The total number of pulmonary arteries: control: N/M n = 30; iPAH: N/M n = 36 (CXCL12), n = 44 (Wnt1); C/P n = 22 (CXCL12); n = 16 (Wnt1).

Nestin^+^ cells were rare in pulmonary arteries of control subjects (Fig 1). Nestin^+^ cells resided in the pulmonary vascular lesions of patients with idiopathic PAH (iPAH) (Fig 1). Nestin staining was frequently localized to the ECs lining the vascular channels of plexiform lesions. These ECs were identified by expression of vWF and CD31. Nestin^+^ vWF^+^ and Nestin^+^ CD31^+^ cells were among the most abundant Nestin^+^ cells in the lung vascular lesions of iPAH patients (Fig 1). There was a small trend towards elevation, but no significant increase, in the fraction of Nestin^+^ cells in the non-muscular and muscular pulmonary arteries of iPAH patients compared to control patients (Fig 1). In contrast, we found that the fraction of Nestin^+^ cells was significantly higher in the pulmonary arteries with concentric and plexiform lesions of iPAH patients, compared to muscular and non-muscular pulmonary arteries of both, control and iPAH patients (Fig 1). Similar to the description by Saboor *et al*. [12], we also found Nestin^+^ α-SMA^+^ cells in the pulmonary arteries exhibiting occlusive arteriopathy (Fig 1). Furthermore, CXCL12^+^ Nestin^+^ cells were not significantly different in non-muscular and muscular pulmonary arteries of control and iPAH patients (Fig 2). In concentric and plexiform lesions, CXCL12^+^ Nestin^+^ cells were elevated (Fig 2). We also identified a significant increase in the fraction of Wnt1^+^ Nestin^+^ cells in the muscular and non-muscular pulmonary arteries of iPAH patients, compared to muscular/non-muscular arteries of controls (Fig 2). Wnt1^+^ Nestin^+^ cells were even further elevated in the concentric and plexiform lesions of iPAH patients (Fig 2).

### Nestin expression in the pulmonary arterial lesions of animals with severe PH

**Fig 3:**
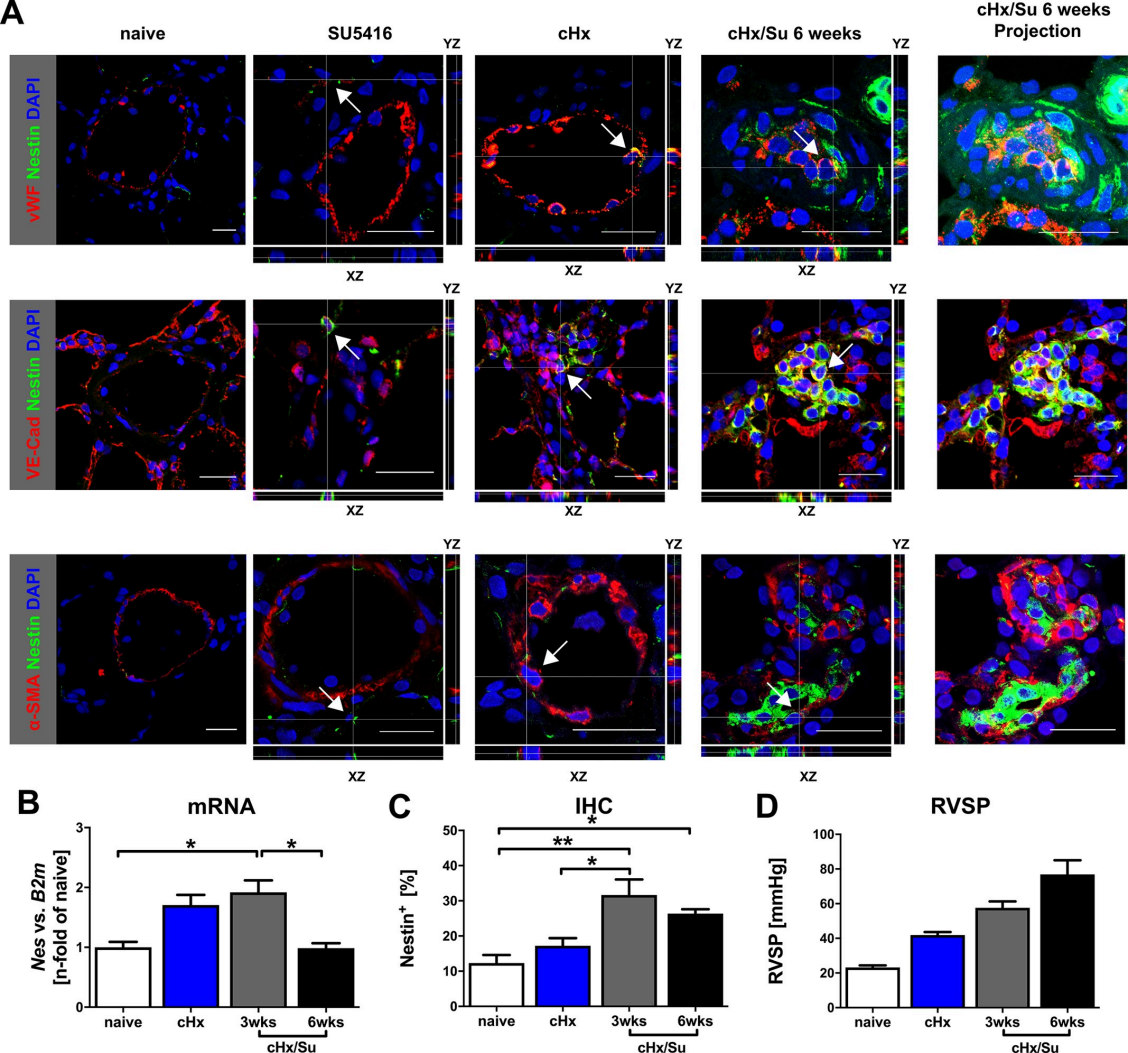
Nestin expression in a rat model of severe PH. (**A**) Representative merged double IF images of optical sections (naïve) and representative orthogonal views of Z-stacks (SU5416, cHx and cHx/Su) obtained by confocal microscopy show the localization of Nestin^+^ cells in pulmonary arteries. Staining further shows expression of endothelial markers vWF and VE-cadherin, or PASMC marker α-SMA. The image on the left shows a representative pulmonary artery of a naïve rat for each staining. On the right side, a projection of the complete Z-stack is shown for the cHx/Su 6 weeks images. Arrows point to representative Nestin^+^ vWF^+^, Nestin^+^ VE-cadherin^+^, and Nestin^+^ α-SMA^+^ cells. The thin white lines show the location of reslicing in X-, Y- and Z-direction. Scale bar: 20 μm (naïve), 25 μm. Nuclear counterstaining with DAPI. Fluorochromes: Nestin (AF488), vWF (AF594), VE-cadherin (AF594), α-SMA (AF594). (**B**) Quantitative RT-PCR of Nes mRNA expression in the lung tissue homogenate of naïve rats, rats exposed to cHx (3 weeks) and the cHx/Su protocol (3 and 6 weeks). (**C**) Quantitative analysis of the fraction of Nestin^+^ cells in pulmonary arteries using immunohistochemistry for Nestin in lung tissue sections from naive rats, rats exposed to cHx (3 weeks) and the cHx/Su protocol (3 and 6 weeks). (**D**) Right ventricular systolic pressure (RVSP) for the different groups confirm PH in cHx and cHx/Su rats. Each bar represents the mean+SEM of n = 3–4 animals. *P<0.05, **P<0.01 (Kruskal-Wallis).

In lungs or pulmonary arteries of naïve animals, Nestin^+^ cells were only occasionally found (Fig 3A, 3B and 3C). A single injection of SU5416 alone did not increase Nestin expression in the lungs or in pulmonary arteries, or promoted PH after 21 days (representative data from 2 rats): the mRNA expression of Nestin was 0.893 and 0.887 (n-fold of naïve control in Fig 3B). The fraction of Nestin^+^ cells in pulmonary arteries using IHC was 6.03% and 11.53% (% of total cells). RVSP was 29.7 and 31.3 (mmHg) and hence only marginally higher than naïve controls (Fig 3D). Yet exposure to cHx and particularly cHx/Su elevated Nestin expression in the lung and pulmonary arteries (Fig 3B and 3C). PH was confirmed using right heart catheterization (Fig 3D). The *Nes* mRNA level returned to the level of naïve rats in 6 weeks cHx/Su rats (2 weeks after cessation of cHx), but a high fraction of Nestin^+^ cells persisted at 6 weeks in the pulmonary arteries of cHx/Su (Fig 3B and 3C). Using double IF stainings, we identified Nestin staining in vWF^+^ and VE-cadherin^+^, but also in α-SMA^+^ cells (Fig 3). Hence, in the rat cHx/Su model, lumen-occluding ECs expressed Nestin, similar to human iPAH.

### Nestin expression in rat and human lung ECs

**Fig 4:**
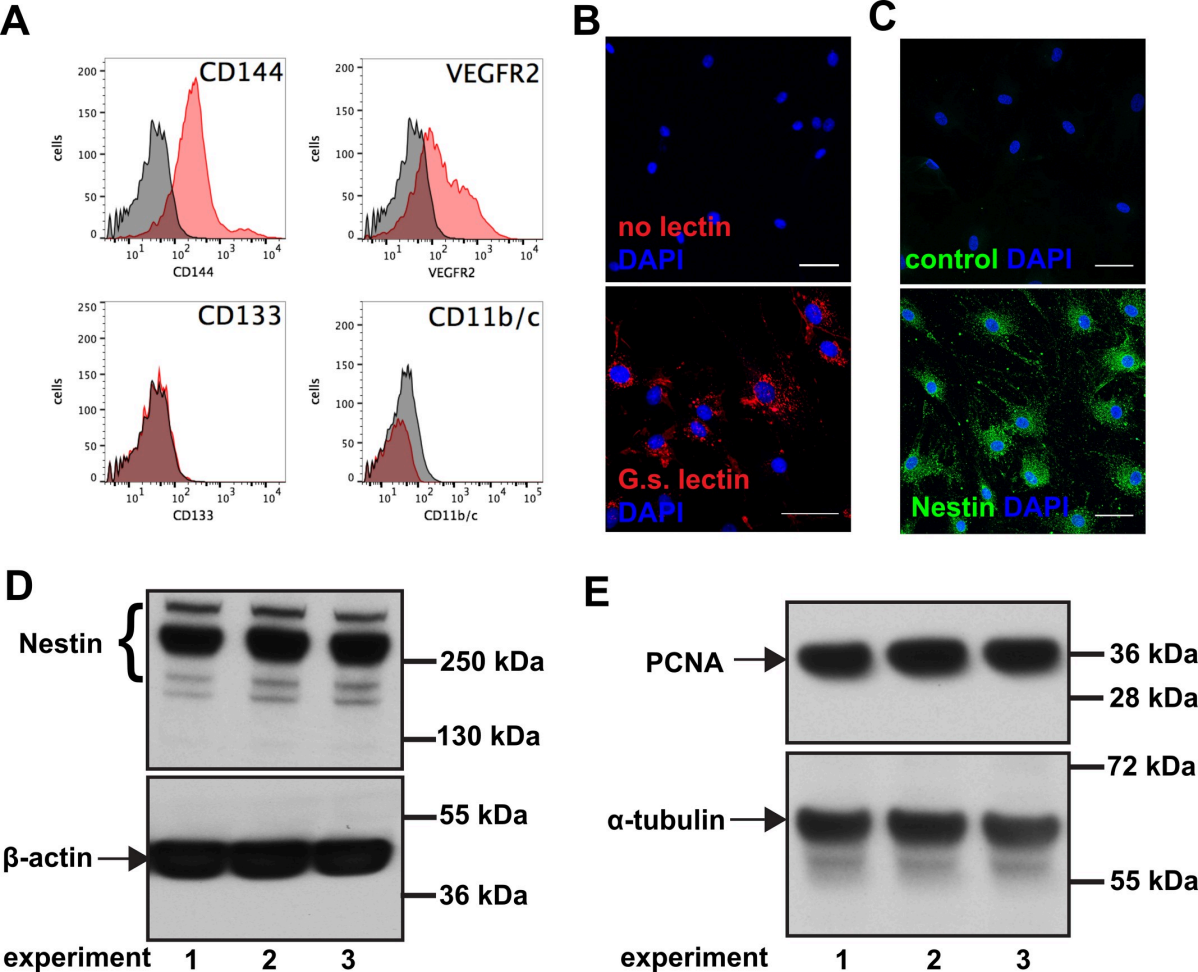
Rat and human lung endothelial cells express Nestin. (**A**) Representative flow cytometry of rat lung ECs for CD144 (Vascular Endothelial-cadherin) and VEGFR2. Rat lung ECs were negative for myeloid/hematopoietic markers CD133 and CD11b/c. The specific antibody staining is red, and the corresponding isotype is grey. (**B**) Rat lung ECs bind *Griffonia simplicifolia* lectin (G.s.), indicating microvascular ECs. (**C**) Rat lung ECs grown on chamber slides express Nestin. Note the perinuclear localization and the filaments extending throughout the cytoplasm. Control means omission of primary antibody. (B-C): Scale bars: 50 μm. (**D**) Representative Western blot showing Nestin expression in HLMVECs (β-actin as loading control). (**E**) Representative Western blots showing PCNA expression in HLMVECs (α-tubulin as loading control). Experiments 1–3 indicate unstimulated cells grown in separate dishes in EGM-2MV for Western blot analysis.

Our hypothesis was that ECs are one important source of Nestin^+^ cells in the lung vascular lesions in PAH. To further support our hypothesis, we first tested physiological expression of Nestin in lung ECs. We isolated ECs from the lung periphery of rats and these ECs expressed the endothelial markers CD144 and VEGFR2. The rat ECs lacked expression of the myeloid and hematopoietic markers CD133 and CD11b/c (Fig 4A). The ECs were microvascular as demonstrated by binding of *Griffonia simplicifolia* lectin (Fig 4B). They further expressed Nestin under proliferating, sub-confluent conditions (Fig 4C). Commercially available HLMVECs also expressed Nestin during cell expansion (Fig 4D and 4E). Hence, Nestin expression is physiologic in proliferating lung ECs.

### Nestin expression and endothelial proliferation

**Fig 5:**
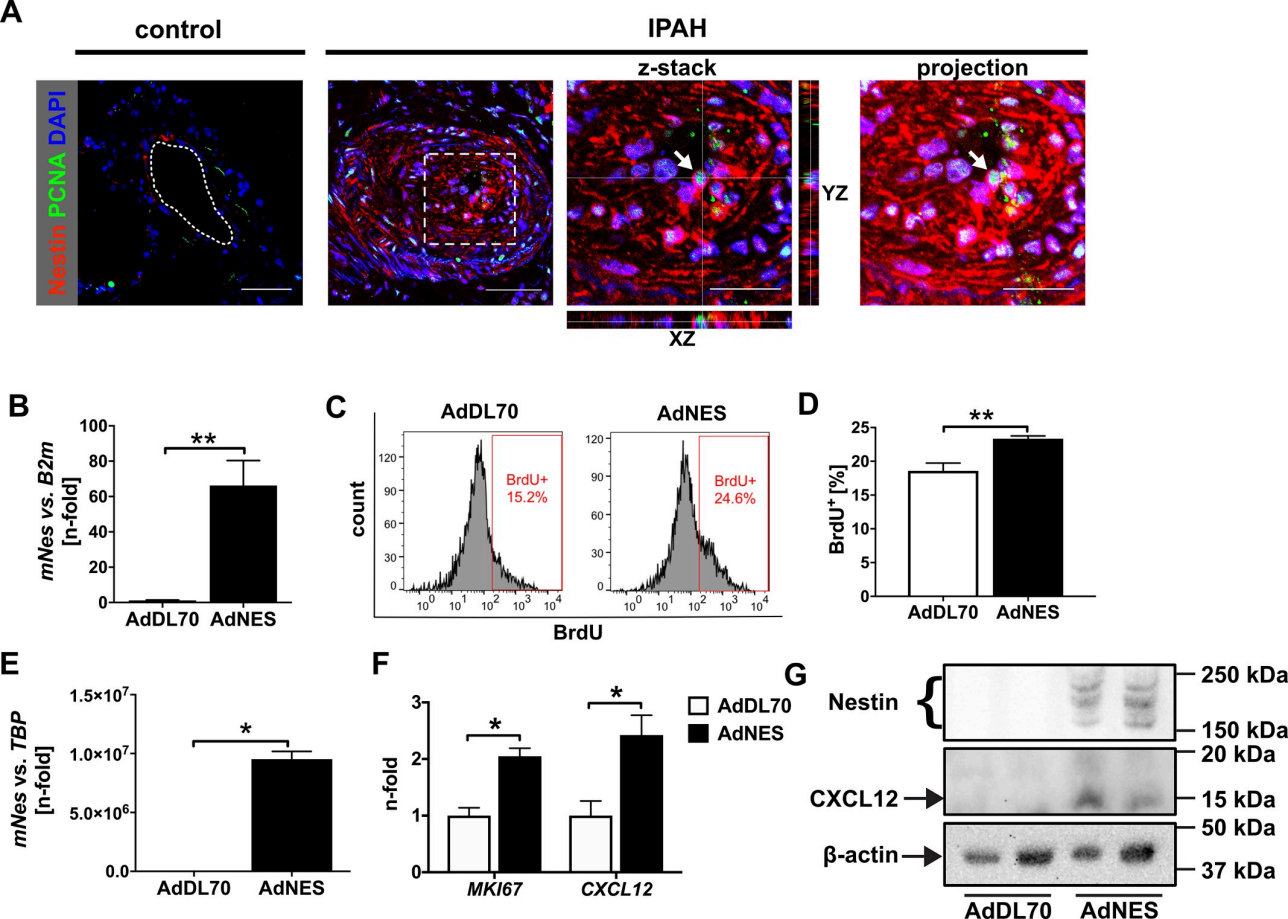
Nestin overexpression promotes endothelial proliferation. (**A**) Co-immunofluorescence staining of PCNA and Nestin reveals multiple Nestin^+^ PCNA^+^ cells (arrow) in the intima of a concentric lesion from a PAH patient. In contrast, no Nestin^+^ PCNA^+^ cells were detected in the intima of a pulmonary artery from a control subject. Scale bars: 50 μm (overview), 25 μm (detail images). For PAH, the image on the left shows an overview of the blood vessel, whereas the images in the middle demonstrate orthogonal views of z-stacks from the area indicate by a dotted box. The image on the right is an intensity projection of the whole z-stack. (**B**) Transient Nestin overexpression in rat lung ECs 72h after adenoviral transduction (qRT-PCR). β2-microglobulin (*B2m*) was used as housekeeping gene. n = 6 per group. (**C-D**) Increased BrdU incorporation over 4 h following Nestin overexpression in rat lung ECs (72h after adenovirus inoculation). (C) Representative histograms and (D) quantification. n = 6 per group. (**E**) Transient Nestin overexpression (qRT-PCR) in human control PAECs 72h after adenovirus transduction (qRT-PCR). TATA-binding protein (*TBP*) was used as housekeeping gene. (n = 4 per group). (**F**) qRT-PCR of *MKI67* (Ki-67, a proliferation marker) and *CXCL12* in hPAECs 72h after AdNES or AdDL70 treatment. n = 4 per group. All data shown as mean+SEM. *P<0.05, **P<0.01 (Mann-Whitney). (**G**) Representative Western blots demonstrating the transgenic Nestin protein expression and elevated CXCL12 protein expression in PAECs 72h after adenoviral transfection with AdNES vs. AdDL70. β-actin was used as loading control.

**Fig 6:**
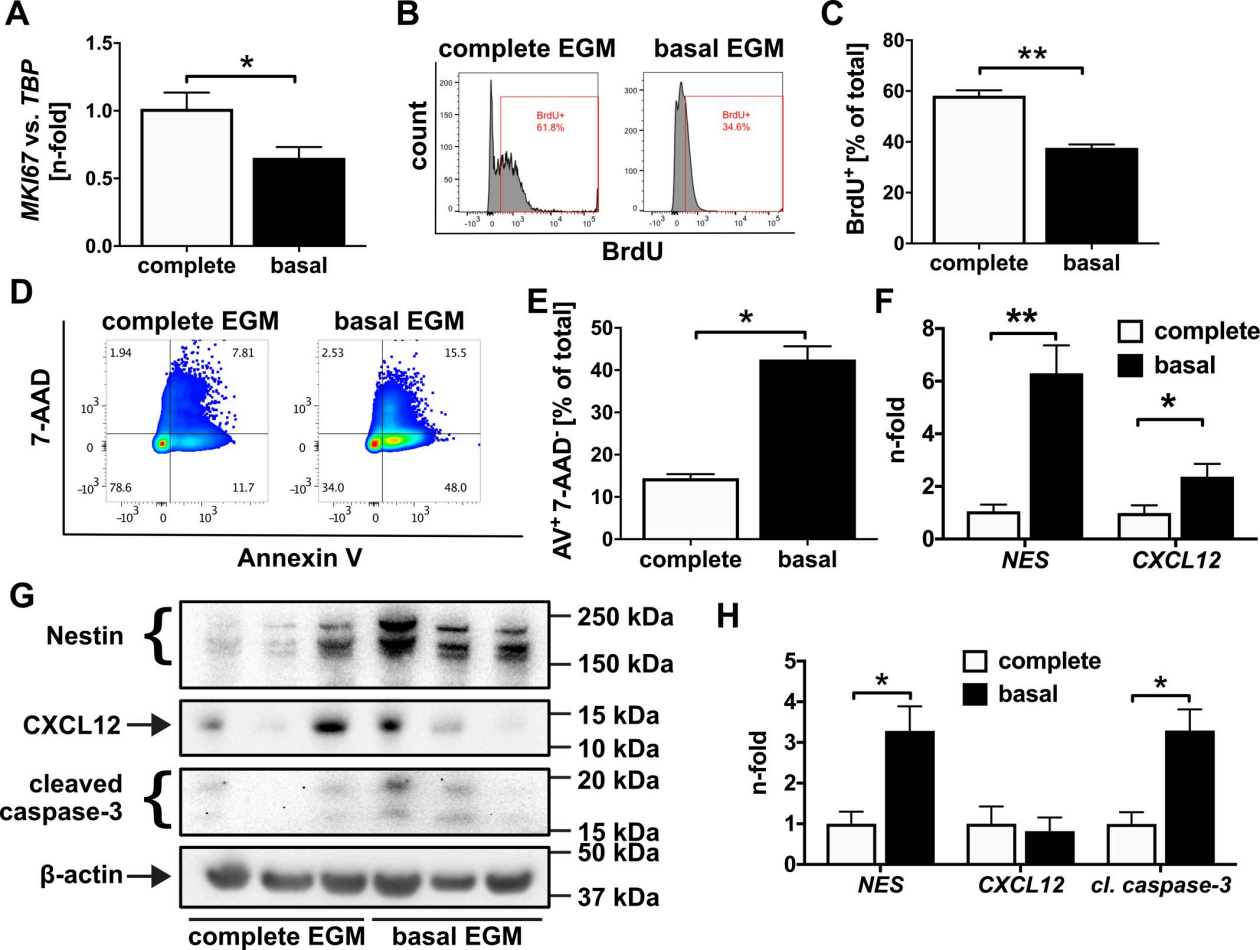
Serum starvation reduces proliferation and induces apoptosis and Nestin expression in human PAECs. (**A**) qRT-PCR expression of *MKI67* (Ki67) in human PAECs cultured for 24h with complete EGM-2 or basal EGM (= serum starvation). (**B-C**) 24h serum starvation reduces proliferation in PAECs. (B) Representative histograms and (C) quantification. (**D-E**) 24h serum starvation promotes apoptosis measured as Annexin V (AV)^+^ 7-aminoactinomycin (7-AAD)^-^ cells using flow cytometry. 7-AAD was added to exclude necrotic cells, which are 7-AAD^+^. (D) Representative dot plots and gating. (E) Quantification. (**F**) qRT-PCR of mRNA expression of *NES* and *CXCL12* after 24h serum starvation. (**G**) Representative Western blots show that serum starvation promotes Nestin protein expression and increases caspase-3 cleavage, but not CXCL12 protein expression. (**H**) Quantification of Western blots in (G). n = 3 per group (H), n = 4 per group (E), n = 6 per group (A, C, F). All data shown as mean+SEM. *P<0.05, **P<0.01 (t-test, Mann-Whitney).

To determine whether Nestin expression is associated with proliferation of intima cells in pulmonary artery lesions of patients with iPAH, we investigated double IF staining for the proliferation marker PCNA and Nestin in pulmonary arteries from patients with iPAH. We found that Nestin^+^ PCNA^+^ cells accumulated in the neointima lesions from patients with iPAH (Fig 5A). To test if overexpression of Nestin promotes EC proliferation, we overexpressed murine Nestin in cultivated rat lung ECs and human PAECs using an adenovirus (AdNES). AdNES-induced Nestin expression was confirmed by specific PCR and Western blot. Indeed, Nestin overexpression enhanced proliferation (BrdU incorporation and *MKI67* mRNA expression) in rat lung ECs and human PAECs (Fig 5). Interestingly, Nestin overexpression promoted expression of *CXCL12* (Fig 5). Serum starvation (basal EGM) reduced proliferation (BrdU incorporation and *MKI67* expression) and promoted apoptosis (Annexin V binding and caspase-3 cleavage), but also surprisingly induced *NES* (Nestin) mRNA and protein expression in PAECs (Fig 6). Whereas serum starvation also induced *CXCL12* mRNA expression, we did not find a consistent induction of CXCL12 protein expression (Fig 6).

### Nestin overexpression promotes angiogenesis *in vitro*

**Fig 7:**
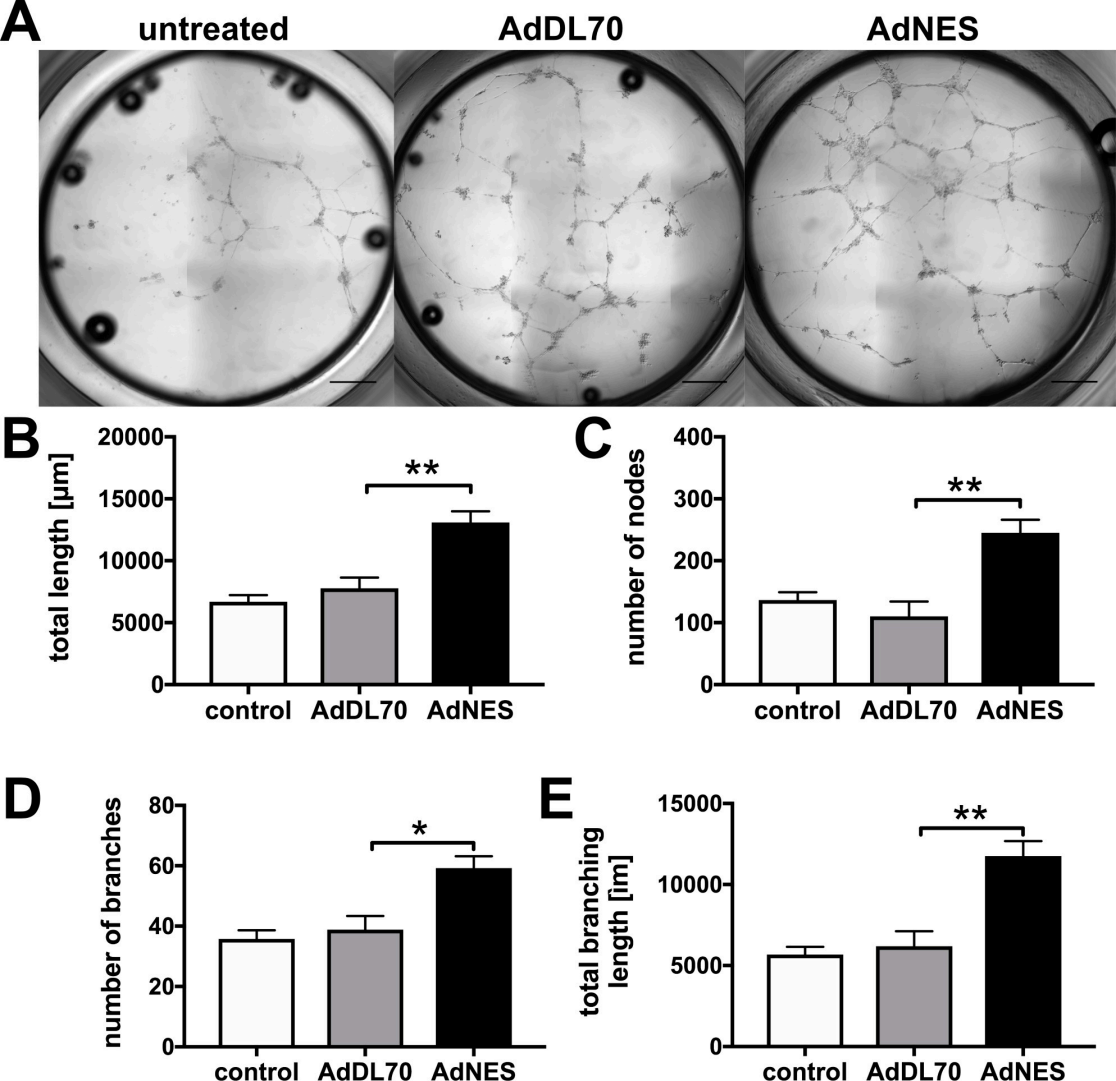
Nestin overexpression promotes angiogenesis in Matrigel. PAECs were treated with AdDL70 or AdNES, or left untreated. After 48h, cells were seeded on Matrigel. (**A**) Representative images of PAECs after 19h on Matrigel in Ibidi μ Angiogenesis plates. Scale bar: 500 μm. Note that the images of whole wells were obtained using the manual stitching function in Olympus CellSens software. (**B-E**) Quantification of total tube length, number of nodes, number of branches and total branching length. Mean+SEM (n = 4–5 per group). *P<0.05, **P<0.01. Statistics were calculated using Mann-Whitney for AdDL70 vs. AdNES.

Because aberrant angiogenesis is a feature of advanced PAH, we tested whether overexpression of Nestin in PAECs promotes angiogenesis in Matrigel assays. We found that Nestin overexpression increased angiogenic tube formation by PAECs, as shown by higher total tube length, number of nodes, number of branches and total branching length (Fig 7).

## Discussion

Despite improved life expectancy, PAH remains a fatal disease [2,3,33] and current treatments fail to target the proliferation, which leads to the progressive, occlusive pulmonary arteriopathy [2,33]. In these vascular lesions, the cells are both apoptosis-resistant and hyper-proliferative [5,6]. While the cause of abnormal cell growth remains poorly understood, one concept suggests that endothelial apoptosis causes clonal selection of endothelial-like stem cells, and these stem cells then give rise to apoptosis-resistant, hyperproliferative ECs [8]. The expression of stem cell markers in the lung vascular lesions supports this concept [10–12]. One of these stem cell markers is the type VI intermediate filament protein Nestin, which has been recently implicated in the pathogenesis of PAH [12]. Hence, we hypothesized that Nestin is expressed in ECs from PAH vascular lesions and that Nestin expression contributes to aberrant proliferation and angiogenesis in these ECs.

The main findings in our manuscript are: 1) Nestin is expressed in ECs in the complex lung vascular lesions of PAH patients and cHx/Su rats. 2) Nestin^+^ cells stain for mediators of angiogenesis CXCL12 and Wnt1. 3) Nestin expression is detected in expanding human and rat lung microvascular ECs. 4) Adenovirus-mediated overexpression of Nestin promotes expression of CXCL12, proliferation and angiogenesis in human PAECs. 5) Growth arrest *via* serum starvation induces apoptosis and expression of Nestin.

This is, to our knowledge, the first study using confocal microscopy analysis to reveal that aberrant endothelium is a source of Nestin expression in PAH. In control lungs without evidence for pulmonary vascular disease, Nestin^+^ cells were rarely detected in and around pulmonary arteries. These findings are supported by previous studies showing that Nestin expression is a marker of angiogenic, proliferating endothelium, but not of quiescent ECs [16,22]. Previous work has shown that a fraction of Nestin^+^ cells belongs to the mesenchymal lineage [12]. Such Nestin^+^ α-SMA^+^ cells have been described in remodeled pulmonary arteries from PAH patients [12] and may also represent precursors of adventitia fibroblasts which contribute to PAH pathobiology [34]. To follow the fate of Nestin^+^ cells over time in lung vascular lesions, we evaluated rats with cHx/Su-induced PH. This model is a valid model of severe PH with occlusive lung vascular lesions similar to human PAH [35,36]. We found elevated endothelial Nestin expression in occluded pulmonary arteries from these cHx/Su rats, similar to our results with tissue from human PAH patients. Using IF stainings, we have localized the endothelial Nestin expression mainly to the concentric and plexiform lesions in PAH patients. These findings are consistent with the expression of stem/progenitor cell markers in these lesions in PAH patients [10,12]. It is of interest that we detected a discrepancy between lower lung tissue mRNA expression and higher protein expression of Nestin in pulmonary arteries from rats with established cHx/Su-induced PH. There are several possible explanations for these differences: First, lung tissue expression of Nestin may not adequately reflect the level in the pulmonary arteries, which is more relevant for PH. Second, this discrepancy may also point towards post-translational dynamics in Nestin protein organization and turnover. For example, Nestin protein requires association with other intermediate filament proteins, such as vimentin, to generate functional intermediate filaments [37,38]. Once assembled, intermediate filaments undergo further post-translational modification, such as phosphorylation and glycosylation, which may affect filament turnover [37,38].

To further understand the relationship between Nestin expression and aberrant endothelial proliferation and angiogenesis, we evaluated the ability of Nestin^+^ cells to express angiogenic factors. We found CXCL12 expression in Nestin^+^ cells in PAH vascular lesions. This is relevant, because CXCL12 and its receptor CXC chemokine receptor 4 (CXCR4) are present in complex lesions in PAH [10,11] and CXCL12 promotes aberrant angiogenesis, endothelial proliferation and PH [23,24,39–44] while others have found Wnt1 to be protective from aberrant tumor angiogenesis [45].

To test whether increased Nestin expression promotes endothelial proliferation, we first confirmed that Nestin is expressed during physiologic expansion of rat and human lung endothelium *in vitro*. Further experiments revealed that transient overexpression of Nestin increased endothelial proliferation and angiogenesis *in vitro*. While previous publications found that Nestin expression is common in proliferating and angiogenic ECs [17,21,46,47], our study is the first to prove that Nestin overexpression directly promotes endothelial proliferation and angiogenesis. The literature remains speculative whether Nestin expression is a response to cell proliferation or a driver of cell proliferation [15–17,48,49]. This controversy of Nestin expression and cell cycle progression is further enriched by our finding that reducing proliferation in ECs by serum starvation promoted expression of Nestin. While this may seem counterintuitive at first glance, one explanation is that serum starvation exerts selection pressure via endothelial apoptosis [50], which causes expansion of progenitor-like ECs with higher expression of Nestin. There are precedents for this concept of endothelial injury with expansion of a progenitor-like cell population in systemic hypertension and following apoptosis in HLMVECs [51,52]. Alternatively, upregulation of Nestin may represent a protective mechanism in ECs that also occurs during regular angiogenesis [53]. It is interesting that we further detected a discrepancy in upregulation of CXCL12 mRNA and the lack of upregulation of CXCL12 on a protein level in serum starved ECs despite Nestin upregulation. One potential explanation for this discrepancy is a selective inhibition of translation during apoptosis [54]. The reason why CXCL12 translation but not Nestin translation was inhibited requires further evaluation that is beyond the scope of the current manuscript.

While the detailed mechanism of proliferation due to elevated Nestin expression is unclear and remains to be addressed in future studies, our results show a strong association between Nestin overexpression and upregulation of CXCL12 in a non-apoptotic environment. We acknowledge recent work showing that Nestin contributes to activation of other cell growth pathways such as Akt or glycogen synthase kinase 3β and promotes vascular endothelial growth factor-mediated effects [20,53,55]. Likewise, Nestin fosters migration and angiogenesis *via* activation of matrix metalloproteinases 2 and 9 [55].

Surprisingly, it is still unresolved how Nestin upregulation occurs in PAH. Although this intriguing question exceeds the scope of our current study, several potential mechanisms were described in the literature: For instance, fibroblast growth factor 2, which contributes to the aberrant phenotype of PAH endothelium, has been shown to induce Nestin expression [56,57]. Alternatively, the loss of p53 (a repressor of Nestin transcription) could promote Nestin expression, and PAH [26,58,59]. Now that a connection between Nestin and PAH pathophysiology has been found in human and animal lung tissue, further evaluation into this pathway will be essential to our understanding of PAH.

There are potential limitation to our study which we wish to acknowledge: 1. Our animal experiments used only male rats, which may limit clinical translation of our findings to PAH due to the predominance of female PAH patients. 2. Although our *in vitro* data show that Nestin overexpression promotes endothelial proliferation and angiogenesis, we have not demonstrated whether knock-down of Nestin would indeed inhibit endothelial proliferation and angiogenesis.

In conclusion, our data suggest that in PAH, the abnormal ECs undergoing aberrant proliferation and angiogenesis in the lung vascular lesions are a source of Nestin expression. Increased Nestin expression contributes to uncontrolled proliferation and angiogenesis in these ECs. One potential mechanism of aberrant angiogenesis and proliferation in Nestin^+^ ECs is increased expression of the angiogenic factor CXCL12. Additional studies are required to answer the question whether targeting Nestin is a promising approach to alter the aberrant endothelial function that marks PAH.
